# Comparison of Abnormal Cervical Cytology from HIV Positive Women, Female Sex Workers and General Population

**Published:** 2015-04

**Authors:** Homeira Vafaei, Nasrin Asadi, Leila Foroughinia, Alireza Salehi, Safieh Kuhnavard, Mojgan Akbarzadeh, Hamid Reza Ravanbod, Ferdos Mohamadalian, Maryam Kasraeian

**Affiliations:** 1 Maternal Fetal Medicine Research Center, Shiraz University of Medical Sciences, Shiraz, Iran;; 2 Department of Obstetrics and Gynecology, Shiraz University of Medical Sciences, Shiraz, Iran;; 3 Research Center for Traditional Medicine and History of Medicine, Shiraz University of Medical Sciences, Shiraz, Iran;; 4 Department of Pathology, Shiraz University of Medical Sciences, Shiraz, Iran;; 5 Biomedical Science Centre, Charles Sturt University, Melbourne, Australia;; 6 Nursing School and Midwifery, Shiraz University of Medical Sciences, Shiraz, Iran

**Keywords:** HIV/AIDs, Sex Worker, Pap smear, Iran

## Abstract

**Background:**

Sex workers and HIV seropositive women are at high risk of abnormal cervical cytology. The objective of this study was to compare the cervical cytology among three groups of women: active sex workers, HIV-infected women, and general population in Iran.

**Methods:**

This was a cross-sectional study performed in Hazrat Zeinab, Lavan clinics and drop in center (DIC) in Shiraz, Iran. This study was performed from October 2009 to October 2011. A total of 266 patients were assigned into three groups: sex-workers (85), HIV positive patients (100), and general population (81). Pap smear was performed for all participants from the exocervix and endocervix, using a plastic Ayres’s spatula and cytobrush. The samples were sent to a pathology center, using a liquid-based media.

**Results:**

The risk of cervical infection in sex workers and HIV positive women was greater than the general population (OR=5.47, 95% confidence interval [CI]:2.24, 13.40), (OR=3.71, 95% CI:1.52, 9.09), respectively. The frequency of abnormal cervical cytology in the HIV positive and sex worker groups was higher than the general population (OR=6. 76, 95% CI:2.25, 20.32), (OR=3. 80, 95% CI:1.19, 12.07), respectively. Low-grade squamous intraepithelial lesion (LSIL) and high-grade squamous intraepithelial lesion (HSIL) were associated with CD4 cell count<200Í106/L, P=0.021 and P<0.001, respectively.

**Conclusion:**

Vaginal infections were seen more often in the sex worker group, and abnormal cervical cytology was greater in the HIV positive group.

## Introduction

Compared to general population, sex workers and HIV seropositive women are at high risk for abnormal cervical cytology.^1^^-^^7^ The prevalence of squamous intraepithelial lesions (SIL) in HIV positive group varies from one study to another. In one study, 7.17% of the seropositive women have SIL, which is two folds compared to general population, and in another study it was three fold.^1^^,^^2^ Women infected with HIV who have Low-grade squamous intraepithelial lesion (LSIL) are at risk of progression to high-grade squamous intraepithelial lesion (HSIL) and invasive carcinoma.^3^ The prevalence of abnormal cervical cytology in sex workers is higher than general population (10.12% vs. 3.92%).^4^

Cervical cancer following cervical dysplasia is a major health concern. Despite decline in its prevalence and mortality, cervical cancer is still the third most common cause of cancer death among women worldwide.^8^ In a systematic review published in Iran in 2012, the mean cervical cancer age-standardized incidence was 2.5 per 100 000 in pathology-based cancer registries, and 6 per 100 000 in population-based registries. Although the incidence of cervical cancer is low in Iran, the mortality to incidence ratio was reported to be over 42%. In another study in Iran was showed that HPV was detected in 90.8% patients with abnormal cervical cytology.^9^^-^^11^

There are several risk factors associated with cervical cancer, including race, parity, smoking, the use of oral contraceptives, age of first sexual intercourse, multiple and new partners, immune deficiency, and low socioeconomic status.^12^^-^^14^ In many societies, due to a range of social and legal discrimination issues, sex workers are usually poorly identified and do not have regular Pap smears or other routine health checkups. In recent years in Iran, voluntary counseling and testing (VCT) centers have been offering services to women at high risk due to their sexual behavior; however, a large number of them still have not been identified.

Human immunodeficiency virus (HIV) seropositive patients are another high risk group for cervical cancer.^15^^,^^16^ Despite several published papers about cervical dysplasia in both HIV-infected women and sex workers in the literature, this study is unique because it compares these two groups with general population. Shiraz is a large city and a referral center in the southern part of Iran. To our knowledge, the present Pap smear study in sex workers and HIV groups, in Shiraz City, Iran is the first study in this category. Therefore, this study will be helpful for future health planning.

## Materials and Methods

*Study Population*

This was a cross-sectional study undertaken in Hazrat Zeinab, Lavan clinics and drop in center (DIC) in Shiraz, Iran. All sex workers and HIV Positive women who registered in these centers during the period of the study were included. The Lavan clinic and DIC provide free health facilities for sex-workers and HIV positive patients, such as Pap smears, contraception and investigation of sexually transmitted diseases (STD). This study was performed during October 2009 and October 2011. The Institutional Review Board (IRB) of Shiraz University of Medical Sciences approved the study protocol and approval of the Ethics Committee was obtained before beginning of the study. All patients signed the informed consent.

We used convenience sampling. Three groups of participants were included in this study. The first group was 100 HIV–seropositive women. In this group, the patients were adult (>18 years of age) and had two positive HIV tests (the enzyme-linked immunosorbentassay (ELISA) and the western blot). The second group included 85 sex workers. Patients in this group were adult (>18 years) and were defined through their history which indicated economic gain from their sexual relationships. The third group (general population) of participants consisted of 81 healthy married women, who attended Hazrat Zeinab clinic for routine workup and regular Pap smear. They had only one partner with negative history of other risk factors such as using illicit drugs. Exclusion criteria included pregnant women, women with history of cervical dysplasia or malignancy, and history of co-morbidities including diabetes mellitus, hypertension or autoimmune disease. 

*Study Protocol*

Demographic data, such as age, parity, marital and educational status, smoking frequency, drug consumption habits, number of sexual partners, contraception, age at first intercourse, and history of post -coital bleeding were recorded. Pap smear was done for all participants from the exocervix and endocervix using a plastic Ayres’s spatula and cytobrush and the specimens were sent to a pathology center  in a liquid-based media. In contrast to what is usually done in Pap smear where the sample is spread directly onto a microscope, we separated the head of the spatula and put it in a container with the preservative solution or directly rinsed it into the preservative. Then a thin layer of the cells was spread  onto a slide and examined in the usual way under a microscope by the same pathologist. The results were reported according to the Bethesda 2001 system.^17^ HIV-seropositive women also underwent venipuncture, and 5ml venous blood sample was sent to determine their CD_4_+ T-lymphocyte count.

*Statistical Analysis*

Fonn et al. found out that the prevalence of abnormal Pap smear in HIV seropositive women and in general population was 36% and 10.3%, respectively. Accordingly, sample size was calculated to be 40 subjects in this group with alpha 0.05 and power 0.8.^5^

Leung et al. revealed that the prevalence of abnormal Pap smear in sex workers and general population was 12.46% and 4.52%, respectively. Thus, sample size was calculated to be 27 subjects in this group with alpha 0.05 and power 0.8.^4^

The statistical package for social sciences, version 16.0 (SPSS, Chicago, IL, USA) was used for the data analysis. A P value less than 0.05 was considered statistically significant. Data were reported as mean±SD and proportion as appropriate. Univariate statistical analyses were conducted by means of Chi-square and Mann-Whitney tests to compare Pap smear findings and CD_4_+ T-lymphocyte counts as categorical (cut-off value: 200×10^6^/L) and continuous variables, respectively. Multiple analysis was conducted for evaluation of the effect of the risk factors on vaginal infections and cervical cytology findings by logistic regression model.

## Results

Overall, the subjects in each group included 100 HIV-seropositive women, 85 sex workers and 81 general populations. The mean age of the study participants was 36 years old in HIV-seropositive women and general population and 33 years old in sex workers. The marital status in the women is showed in Table 1. 97.5% in the general population group, 67% in the HIV group and 52.9% in the sex worker group were married. The educational status in sex workers and HIV seropositive women was mostly limited to elementary, primary and high school education due to the women’s low socioeconomic status in this study. Addiction to drugs was higher among the sex workers than in the HIV and general population groups and it reached 61%. Contraception methods were also compared in these groups; 17% of the HIV group and 8% of the sex workers did not use contraception at all. Only 58.0% of the HIV group used condoms in their relationships. Fifty five percent of HIV seropositive women had one partner, 20% had two partners and 25% had more than 2 partners. Eighty seven percent of HIV group, 89.5% of sex workers and 85.2% of general population were multiparous. Other factors, such as age at first intercourse, number of partners, parity, and history of post-coital bleeding, are shown in Table 1.

**Table 1:** Demographic and characteristics of HIV-seropositive, sex workers and healthy subjects

	**HIV-positive** **(n=100)**	**Sex-workers (n=85)**	**General- Population (n=81)**	**P value**
Age (years)	36.27 (SD: 8.14)	33.21 (SD: 8.64)	35.78 (SD: 10.93)	0.063
Marital status				0.001
Married (%)	67 (67.0%)	45 (52.9%)	79 (97.5%)	
Single (%)	0 (0.0%)	5 (5.9%)	0 (0.0%)	
Widowed (%)	13 (13.0%)	8 (9.4%)	2 (2.5%)	
Divorced (%)	20 (20.0%)	27 (31.8%)	0 (0.0%)	
Education				0.001
Illiterate (%)	14 (14.0%)	7 (8.2%)	13 (16.0%)	
Primary and high school (%)	59 (59.0%)	63 (74.1%)	33 (40.8%)	
Diploma or higher (%)	27 (27.0%)	15 (17.7%)	35 (43.2%)	
Drug consumption				0.001
Nothing (%)	60 (60.0%)	33 (38.8%)	78 (96.3%)	
Cigarette (%)	20 (20.0%)	10 (11.8%)	3 (3.7%)	
Injection (%)	4 (4.0%)	1 (1.2%)	0 (0.0%)	
Opium (%)	2 (2.0%)	20 (23.50%)	0 (0.0%)	
Methadone (%)	2 (2.0%)	5 (5.9%)	0 (0.0%)	
Others (%)	12 (12.0%)	16 (18.80%)	0 (0.0%)	
Age at 1^st^ intercourse				0.001
≤16 years (%)	39 (39.0%)	32 (37.6%)	30 (37.0%)	
>16 years (%)	61 (61.0%)	53 (62.4%)	51 (63.0%)	
Contraception				0.545
No Contraception (%)	17 (17.0%)	7 (8.2%)	22 (27.2%)	
Condom (%)	58 (58.0%)	37 (43.5%)	9 (11.1.0%)	
Tubal ligation (%)	9 (9.0%)	6 (7.1%)	16 (19.8.0%)	
Other	16 (16.0%)	35 (41.2%)	34 (41.9%)	
Post coital bleeding				0.545
No	93 (93%)	82 (96.5%)	77 (95.1%)	
Yes	7 (7%)	3 (3.5%)	4 (4.9%)	

The risk of cervical infection in sex workers and the HIV positive groups was higher than that in the general population (OR=5.47, 95% confidence interval [CI]:2.24, 13.40), (OR=3.71, 95% CI: 1.52, 9.09), respectively. The adjusted risk of cervical infection (according to age, education, recreational drug use, age at first intercourse, number of partners) in HIV positive and sex workers was also higher than that in the general population (OR=3.66, 95% CI:1.47, 9.13), (OR=5.14, 95% CI:2.03, 13.01), respectively (Table 2). Bacterial vaginosis (Gardenella) was the infection reported most often, followed by Trichomonas vaginalis and Candida albicans. There was no significant association between cervical infection and age at first intercourse (P=0.969), contraception method (P=0.468), bleeding after coitus (P=0.978), number of partners (P=0.775), educational status (P=0.231), or drug abuse (P=0.239). Table 3 displays the frequency of different vaginal infections in the three study groups.

**Table 2:** Multiple analysis of factors affecting cervical infection and abnormal cervical cytology

**Variable**		**Frequency**	**Odds Ratio & ** **95% Confidence Interval (Cervical Infection)**	**P value**	**Odds Ratio &95% Confidence Interval** **(Abnormal Cervical Cytology)**	**P value**
Groups of Study	Normal Population	81				
	HIV Positive	98	4.513 (1.659-12.729)	0.003	4.42 (1.39-14.05)	0.006
	Sex Worker	85	8.054 (2.239-28.965)	0.001	2.04 (0.57-7.27)	0.127
Age			0.995 (0.960-1.032)	0.796	1.053 (1.008-1.099)	0.019
Age at first Intercourse	<16 Years					
	>16 Years		0.880 (0.450-1.700)	0.704	0.716 (0.315-1.625)	0.704
Education	Illiterate	33				
	Under Diploma	154	0.473 (0.189-1.181)	0.109	4.423 (0.873-2.419)	0.073
	Diploma or more	77	0.211 (0.067-0.666)	0.008	7.012 (1.236-9.783)	0.028
Drug Use	No	169				
	Yes	95	1.004 (0.503-2.005)	0.991	2.907 (1.252-6.753)	0.013
Number of Partner	One	137				
	Two or more	127	1.699 (0.716-4.035)	0.229	1.325 (0.504-3.484)	0.569

**Table 3:** Prevalence of vaginal infections

	**HIV-seropositive** **(n=100)**	**Sex workers** **(n=85)**	**General- Population (n=81)**	**P value**
No infection (%)	74 (74.0%)	56 (65.9%)	74 (91.3%)	0.001
Gardenella (%)	10 (10.0%)	19 (22.4%)	0 (0.0%)	
Trichomonas vaginalis (%)	9 (9.0%)	7 (8.2%)	3 (3.7%)	
Candida albicans (%)	7 (7.0%)	3 (3.5%)	4 (5.0%)	

*Cervical Cytology Findings*

The frequency of abnormal cervical cytology in HIV positive women and sex workers was greater than that in the general population (OR=6. 76, 95% CI: 2.25, 20.32), (OR=3. 80, 95% CI: 1.19, 12.07), respectively. Adjusted risk of abnormal cervical cytology (adjusted for age, education, drug abuse, age at first intercourse, number of partners) in HIV positive subjects was also higher than that in the general population (OR=4.42, 95% CI: 1.39, 14.05). (Table 2) However, in the sex worker group, there was no significant statistical difference after adjustment, but there was a clinical difference between them (OR=2.04, 95% CI: 0.57, 7.27). Overall, the frequency of abnormal cervical cytology was 26% in the HIV-seropositive patients, 16.5% in the sex workers, and 4.9% in the general population group.

Univariate analysis was performed in order to determine the risk factors for cervical cytology abnormalities in the HIV-seropositive patients. Overall, 74.2% had CD_4_+ T-lymphocyte counts (CD4)>200×10^6^/L, while 25.8% had CD_4_ cell counts <200×10^6^/L. In the HIV positive group, there was no significant correlation between the number of partners (P=0.797) and parity with cervical cytology, but an apparent association between cervical cytology and drug use was noticed (P=0.02). In this group, also, there was no relationship between the use of barrier method (condoms) and cervical infection (P=0.481).

CD_4_ cell count<200×10^6^/L P<0.001), addiction and smoking (P=0.02) were the strongest predictors of epithelial cell abnormalities in HIV-seropositive women. Both LSIL and HSIL were significantly associated with a CD_4_ cell count<200×10^6^/L (P=0.021, P<0.001).

Univariate analysis was also carried out to determine the risk factors for cervical cytology abnormalities in sex workers. A relationship was not found between the aforementioned risk factors and abnormal cervical cytology and vaginal infection in this study. In other words, risk factors such as drug use (P=0.794), parity (P=0.341), age at first intercourse, post-coital bleeding, number of partners, and educational status were not associated with abnormal cervical cytology and vaginal infections in this group. However, protected intercourse (using  condoms) was found to decrease vaginal infection rate significantly, (P=0.019). (Table 4).

**Table 4:** Results of the Pap smears

**Pap smears**	**HIV-seropositive** **(n=100)**	**Sex workers** **(n=85)**	**General- Population (n=81)**	**P value**
Normal range (%)	74 (74.0%)	71 (83.5%)	77 (95.1%)	<0.001
ASCUS (%)	6 (6.0%)	3 (3.5%)	1 (1.2%)	
LSIL (%)	13 (13.0%)	10 (11.8%)	3 (3.7%)	
HSIL (%)	7 (7.0%)	1 (1.2%)	0 (0.0%)	

## Discussion

Although the incidence rate of cervical cancer in Iran is low, the mortality to incidence ratio is higher when compared to developed countries.^10^ HPV vaccination is not still routinely performed in Iran. Therefore, this screening program, particularly in high risk groups, is very important.

HIV positive patients are considered to be a high risk group for developing cervical cancer and there are many studies discussing the prevalence of cervical dysplasia in this group.^2^ In a large cohort study, Ahdieh et al. investigated the effects of HIV in the course of HPV infections at four American centers. The study showed that HIV infection increased the rate of HPV persistence. In other words, immunosuppression plays a significant role in modulating the natural history of HPV infections. In the same study, the HIV-positive group reported a lower number of recent sex partners, compared to HIV negative group; this indicates that the higher incidence rates of cervical dysplasia were not just associated with higher HPV exposure.^15^ Also, the number of partners and age at first intercourse were not associated with cervical dysplasia in HIV-seropositive patients; this is similar to the observations by another study.^15^

The prevalence of abnormal cervical cytology in HIV positive group from this study was found to be 26%, which is a sixfold increased risk as compared to the general population. Prabha Devi found that 7.17% of HIV seropositive patients had abnormal pap smear.^1^ Fonn showed that the prevalence of abnormal cervical cytology in general population was 4.2%, which is similar to our study.^5^ The risk of abnormal cytology in our study was significantly higher in HIV positive groups compared to general population, which is comparable to the finding of other studies.^1^^,^^6^

In another cohort study undertaken in Brazil, 898 HIV-positive women were studied over a 10 year period, and the prevalence and incidence of cervical squamous intraepithelial lesions (SIL) were associated with the severity of HIV.^3^ The study revealed that CD4 counts ≤200 cells/mm and higher viral load counts were related to SIL incidence. Six et al. also reported a higher SIL prevalence in HIV positive patients in comparison to non-infected women (26.5 versus 7.5%). They showed that the mere immunodeficient HIV-positive status increased the risk for dysplasia, irrespective of CD_4_+ count.^18^

Our findings are in accordance with the above studies. In addition, patients with normal cervical cytology had significantly higher CD_4_+ cell counts and a lower frequency of addiction and smoking. This finding clearly indicates the connection between cervical dysplasia and immunosuppression. In this group, confounding factors, such as smoking and addiction, which are risk factors for SIL were also considered. After adjusting for confounding factors, we found that the HIV-seropositive patients still had higher rates of cervical dysplasia. That’s why some studies have emphasized the importance of performing baseline colposcopy in all cytological abnormalities in this group because of higher prevalence of abnormal cervical cytology and shorter time for progression to carcinoma.^3^^,^^19^ In addition, this procedure is inexpensive and available in our country.

Sex workers are another group of women who are at increased risk of cervical epithelial cell abnormalities. Risk factors such as a high number of sexual partners and low socioeconomic status in this group can affect the results significantly.^20^^-^^21^ Our results showed that 16.5% of sex workers had abnormal cytology. In one Korean study, the prevalence of HPV infection in asymptomatic commercial sex workers was reported to be as high as 83.5%.^22^ This high prevalence of HPV resulted in a higher rate of positive cervical cytology and dysplasia.^21^ In a recent study in Hong Kong, Pap smears were performed for 2697 female sex workers and they showed a significantly higher prevalence of abnormality compared to the control group (12.46% versus 4.52%).^4^ Also, smoking and drug addiction were found to be associated with cervical epithelial cell abnormalities just as in the HIV-seropositive patients. However, these factors were found to have no correlation with cervical cytology abnormalities among sex workers.

According to the results of this study, it is very important to provide more facilities in order to identify these target groups and to adopt prevention strategies for screening and early diagnosis of sexually transmitted disease and cervical cancer. Shorter intervals between performing Pap smears may be considered in these groups, but further studies in larger populations are recommended to be performed to identify the exact interval timing for performing Pap smears.

There were some limitations in this study. The first one is the small population size. The results of this study would be significantly more reliable with a larger studied sample, but it had been limited to registered sex workers and HIV positive patients who attended related clinics. Second, we did not investigate the prevalence of HPV and its subtypes in the three study groups due to financial limitations. Third, not all participants in the third group (general population) were screened for HIV. Despite these limitations, to the best of our knowledge, this is the first study comparing cervical cytology of these groups in Iran. 

## Conclusion

Vaginal infections were seen more often in the sex worker group, and abnormal cervical cytology was greater in the HIV positive group. These data indicate that patients with HIV need to undergo routine Pap smear examinations and further studies in larger populations should be performed to identify the exact interval timing for performing Pap smears. Using condom during sexual intercourse could decrease the vaginal infection rate; we recommend that it should be used for all high risk groups. 
